# EffEctiveness of Prophylactic fOam dressings in the prevention of saCral pressure injuries in at-risk hospitalised patients: the EEPOC trial

**DOI:** 10.1186/s13063-022-06999-y

**Published:** 2023-01-31

**Authors:** R. M. Walker, W. Chaboyer, M. Cooke, J. A. Whitty, L. Thalib, I. Lockwood, S. Latimer, J. Campbell, R. Probert, B. M. Gillespie

**Affiliations:** 1 NHMRC Centre of Research Excellence in Wiser Wound Care, Menzies Health Institute Queensland, School of Nursing and Midwifery, Griffith University, Division of Surgery, Princess Alexandra Hospital, Metro South Health, Brisbane, Australia , Brisbane, Australia; 2 NHMRC Centre of Research Excellence in Wiser Wound Care, Menzies Health Institute Queensland, School of Nursing and Midwifery, Griffith University, Gold Coast, Australia; 3 School of Nursing and Midwifery, Menzies Health Institute Queensland, Griffith University, Brisbane, Australia; 4 Health Economics Group, Norwich Medical School, Faculty of Medicine and Health Sciences, University of East Anglia, Norwich, UK; 5 Department of Biostatistics, Faculty of Medicine, Istanbul Aydın University, Istanbul, Turkey; 6 Menzies Health Institute Queensland, Griffith University, Gold Coast, Australia; 7 School of Nursing and Midwifery, Menzies Health Institute Queensland, Griffith University & Gold Coast University Hospital, Gold Coast Health, Gold Coast, Australia; 8 NHMRC Centre of Research Excellence in Wiser Wound Care, Menzies Health Institute Queensland, Griffith University, Gold Coast, Australia; 9 Stomal Therapy and Wound Management Department in the Division of Surgery, Princess Alexandra Hospital, Metro South Health, Brisbane, Australia

**Keywords:** Acute care, Nursing, Adults, Patients, Pressure injury/ulcer, Prophylactic dressings, Sacrum, RCT, Healthcare-acquired complications

## Abstract

**Background:**

Prophylactic dressings are increasingly used to prevent pressure injuries in hospitalised patients. However, evidence regarding the effectiveness of these dressings is still emerging. This trial aims to determine the clinical and cost-effectiveness of a prophylactic silicone foam border dressing in preventing sacral pressure injuries in medical-surgical patients.

**Methods:**

This is a multicentre, pragmatic, parallel group, randomised controlled trial. A sample size of 1320 was calculated to have >90% power to detect a 5% difference in the primary outcome at an alpha of 0.05. Adult patients admitted to participating medical-surgical wards are screened for eligibility: ≥18 years, admitted to hospital within the previous 36 h, expected length of stay of ≥24 h, and assessed high risk for hospital-acquired pressure injury. Consenting participants are randomly allocated to either prophylactic silicone foam dressing intervention or usual care without any dressing as the control group via a web-based randomisation service independent of the trial. Patients are enrolled across three Australian hospitals. The primary outcome is the cumulative incidence of patients who develop a sacral pressure injury. Secondary outcomes include the time to sacral pressure injury, incidence of severity (stage) of sacral pressure injury, cost-effectiveness of dressings, and process evaluation. Participant outcomes are assessed daily for up to 14 days by blinded independent outcome assessors using de-identified, digitally modified sacral photographs. Those who develop a sacral pressure injury are followed for an additional 14 days to estimate costs of pressure injury treatment. Analysis of clinical outcomes will be based on intention-to-treat, per-protocol, and sensitivity analyses.

**Discussion:**

This trial aims to provide definitive evidence on the effect prophylactic dressings have on the development of hospital-acquired sacral pressure injuries in medical-surgical patients. A parallel economic evaluation of pressure injury prevention and treatment will enable evidence-informed decisions and policy. The inclusion of a process evaluation will help to explain the contextual factors that may have a bearing on trial results including the acceptability of the dressings to patients and staff. The trial commenced 5 March 2020 and has been significantly delayed due to COVID-19.

**Trial registration:**

ANZCTR ACTRN12619000763145. Prospectively registered on 22 May 2019

## Administrative information

Note: the numbers in curly brackets in this protocol refer to SPIRIT checklist item numbers. The order of the items has been modified to group similar items (see http://www.equator-network.org/reporting-guidelines/spirit-2013-statement-defining-standard-protocol-items-for-clinical-trials/).

**Table:** 

Title {1}	EffEctiveness of Prophylactic fOam dressings in the prevention of saCral pressure injuries in at-risk hospitalised patients: The EEPOC Trial.
Trial registration {2a and 2b}.	Australian New Zealand Clinical Trials Registry: a primary registry in the World Health Organization International Clinical Trials Registry Platform Registry Network: ACTRN12619000763145, prospectively registered 22 May 2019, https://www.anzctr.org.au/TrialSearch.aspx
Protocol version {3}	Version 6, 15/10/2021
Funding {4}	Australian Federal Government National Health and Medica Research (NHMRC) Project Grant APP1158379.
Author details {5a}	^1^NHMRC Centre of Research Excellence in Wiser Wound Care, Menzies Health Institute Queensland, School of Nursing and Midwifery, Griffith University, Division of Surgery, Princess Alexandra Hospital, Metro South Health, Brisbane, Australia r.walker@griffith.edu.au, +61 7 3176 5843, ORCID 0000-0002-6089-8225, Twitter @RachelMWalker ^2^NHMRC Centre of Research Excellence in Wiser Wound Care, Menzies Health Institute Queensland, School of Nursing and Midwifery, Griffith University, Gold Coast, Australia ^3^School of Nursing and Midwifery, Menzies Health Institute Queensland, Griffith University, Brisbane, Australia ^4^Health Economics Group, Norwich Medical School, Faculty of Medicine and Health Sciences, University of East Anglia, Norwich, UK ^5^Department of Biostatistics, Faculty of Medicine, Istanbul Aydın University, Istanbul, Turkey ^6^Menzies Health Institute Queensland, Griffith University, Gold Coast, Australia ^7^School of Nursing and Midwifery, Menzies Health Institute Queensland, Griffith University & Gold Coast University Hospital, Gold Coast Health, Gold Coast, Australia ^8^NHMRC Centre of Research Excellence in Wiser Wound Care, Menzies Health Institute Queensland, Griffith University, Gold Coast, Australia ^9^Stomal Therapy and Wound Management Department in the Division of Surgery, Princess Alexandra Hospital, Metro South Health, Brisbane, Australia ^10^NHMRC Centre of Research Excellence in Wiser Wound Care, Menzies Health Institute Queensland, School of Nursing and Midwifery, Griffith University, Gold Coast, Australia
Name and contact information for the trial sponsor {5b}	Dr Julie Glover, NHMRC 16 Marcus Clarke Street, Canberra ACT 2601 (GPO Box 1421) Canberra ACT 2601, Australia
Role of sponsor {5c}	The NHMRC (sponsor) played no part in study design; collection, management, analysis, and interpretation of data; writing of the report; or the decision to submit the report for publication.

## Introduction

### Background and rationale {6a}

Many hospitalised patients are at high risk of developing pressure injuries (PI). PI onset can be rapid, with injury of skin and/or tissue over bony prominences [1]. Given its anatomical position, the most common location for PI is the sacrum, which is particularly vulnerable to injury and difficult to treat [1]. This is due to pressure and shear that can result from limited mobility and/or long periods of time sitting due to head of bed elevation [1].

A recent meta-analysis found the global prevalence of hospital-acquired pressure injury (HAPI) in adults was 8.4% [2]. HAPI cost approximately US$11 billion in the USA (between the period of 2000 and 2012) (US) [1], £1.4–£2.1 billion in the UK (2011) [3], and in European countries such as the Netherlands up to $2.8 billion annually (USD in 2009) [1]. A 2022 cost analysis of HAPI in Australian public hospitals reported AU $9.11 billion [4]. HAPI have negative consequences for patients in terms of pain [5] and represent a significant economic burden for health services and communities. Therefore, there is increasing emphasis on implementing pressure injury prevention (PIP) strategies. These strategies include risk assessment, support surfaces, nutritional support, repositioning, and moisture and incontinence management, and more recently, prophylactic dressings [6–9]. Preventing HAPI has the potential to save healthcare dollars which can be redirected to other priority areas, free-up hospital beds and improve the overall quality of patient care, outcomes, and experience consistent with national and international priorities.

There is an increasing emphasis on the use of a range of PIP strategies, including the use of prophylactic dressings. These dressings have demonstrated clinical effectiveness in preventing PI in some patient populations and contexts [1, 7, 8]. However, authors of a Cochrane Review updated in 2018 reiterated the certainty of evidence for using dressings in HAPI prevention was low to very low [10]. Since the 2018 Cochrane Review, authors of two high-quality trials of prophylactic silicone dressings have reported positive outcomes but recommend further rigorous testing in clinical practice is needed [11, 12]. Prior to these two studies, rigorous methodological approaches (that is, randomisation, allocation concealment, and blinded outcome assessment via photography) in assessing the feasibility of using prophylactic dressings to prevent PI a general medical-surgical patient population had only been reported in a single pilot study [13].

Cost-effectiveness analyses of prophylactic dressings are sparse. Two within-trial cost-analyses reported prophylactic dressings led to cost savings [14, 15]. The first trial used a retrospective approach to cost analysis that did not include inpatient bed-day costs [15], while the more recent trial calculated an incremental cost-effectiveness ratio based on the average duration of patient admission and expressed as cost per PI avoided [14].

This previous research led us to design a robust randomised controlled trial (RCT) trial to determine the clinical and cost-effectiveness of a prophylactic silicone foam border dressing in preventing sacral HAPI in hospitalised patients assessed at risk for PI. Process evaluation data will also be collected, to evaluate contextual factors (e.g. patient-, provider-, and system-level factors) that may have moderated the effect of an intervention [16].

### Objectives {7}

The primary objectives of the EEPOC trial are to:

1. Determine the clinical and cost-effectiveness of a prophylactic silicone foam border dressing in preventing sacral hospital-acquired pressure injuries (HAPI) in hospitalised patients assessed at risk for PI
2. Describe the characteristics and clinical care received by patients who develop a sacral HAPI up to 14 days, or until hospital discharge or death after PI occurrence
3. Understand patients and staff perspectives on the use of prophylactic dressing

The primary hypothesis is cumulative incidence (expressed as a percentage) of patients who develop sacral HAPI up to 14 days of admission will be less in patients who are randomly allocated to the routine care plus prophylactic silicone foam dressing group (intervention), compared to patients allocated to routine care only (control).

The secondary hypothesis is patients in the prophylactic dressing group (intervention) will have better outcomes compared to the routine care only group (control) including (1) time to onset of sacral HAPI, (2) incidence of severity of sacral HAPI (based on stage), (3) cost-effectiveness of sacral dressings for the prevention of HAPI, and (4) process evaluation.

### Trial design {8}

This study is a multi-site, parallel group, superiority RCT comparing the effectiveness of a prophylactic silicone foam border dressing in preventing sacral HAPI in hospitalised patients assessed at risk for PI. The unit of randomisation is eligible consenting patients who receive either the prophylactic sacral dressing or the routine care alone. Randomised to either the dressing group or the routine care only group is via a computer-generated 1:1 ratio, in varying permuted block sizes of four, six, and eight, stratified by hospital and division (i.e. medical or surgical).

All patients are followed for up to 14 days or until the trial endpoint, which is development of a new sacral HAPI, hospital discharge, or death—whichever occurs first. Patients who develop a HAPI in either arm are exited from the prevention trial and followed for an additional 14 days until discharge or death—whichever occurs first (up to a total of 28 days) to provide data on PI prevention and treatment costs. This will enable an extended economic evaluation that considers the benefits of prevention in terms of any reduced HAPI treatment costs or HLOS, alongside the costs of prevention.

An integral part of this study is its process evaluation. Process evaluations are particularly important for multi-site trials where it is necessary to determine whether interventions are implemented and received consistently across sites and to understand differences between them [17]. They also provide detailed documentation to enable replication, and information regarding the mechanism of action for significant or non-significant effects, thereby enhancing an intervention’s validity [16, 17].

## Methods: participants, interventions, and outcomes

### Study setting {9}

This multi-site RCT is currently taking place at three acute-care public hospitals in Southeast Queensland, Australia. Two of the hospitals are quaternary hospitals and the third, a tertiary facility. All sites have participated in previous clinical trials. For more details, refer to the Australian New Zealand Clinical Trials Registry: ACTRN12619000763145.

Each site is supported by associate investigator (AI) nurse leaders, who provide ongoing clinical expertise and support for the study. We recruit eligible participants from general medical-surgical wards at each site. The inclusion of multiple sites and wards spreads the recruitment burden and allows testing of the intervention in a variety of acute medical and surgical settings to ensure diverse geographical, unit size, and sub-speciality representation.

### Eligibility criteria {10}

A consecutive sample of all eligible at-risk adult patients admitted to the participating medical-surgical wards are invited to participate in the trial by registered nurse research assistants (ResN). Patients who meet the following inclusion criteria are eligible for recruitment into the sample:

Inclusion criteria:

- ≥18 years of age
- Patients assessed as being at-risk of HAPI within medical-surgical settings that have been screened within 36 h of hospital admission. ‘At-risk’ is defined as patients with either a completed PI risk assessment (Waterlow Score, or clinical judgement scores of ‘Yes, at risk’ or ‘No, not at risk’) that scores them at-risk of PI, or limited mobility (where human or resource assistance is needed to move), for those who do not have a completed risk assessment
- Expected hospital length of stay ≥24h following recruitment
- Patient or their proxy able to provide written informed consent

Exclusion criteria:

- Patients who are unable to be turned (e.g. due to unstable spinal injury)
- Patients with existing sacral PI, injury, allergy, or lesion in the sacral area at the time of recruitment
- Patients with urinary and/or faecal incontinence at the time of recruitment
- Patients who are unable to speak or understand English where no interpreter is present at the time of recruitment

### Who will take informed consent? {26a}

ResNs at each site trained in Good Clinical Practice (GCP) screen and recruit participants, obtain their consent, and randomly allocate them to a study group via an independent web-based randomisation service (in accordance with the hospital procedures informed by state governance and international standards). Written informed consent is sought from each patient, family member, or legal guardian (proxy) prior to randomisation.

If consenting participants are allocated to the intervention group, they will be asked to assess their level of satisfaction with comfort of the dressing. They may also be invited to participate in an individual interview conducted by the research assistant to describe their experience of participating in the trial, as part of the process evaluation.

If the participant or their person responsible (i.e. proxy) is unable to read, an impartial witness must be present during the entire consent process. After the patient or their proxy has orally consented to the patient’s participation and has signed the consent form, the witness must also sign and date the consent to attest the information is understood by the patient or their proxy and consent was given freely. Furthermore, where the impairment, disability, or illness is temporary or episodic, an attempt will be made to seek consent at a time when the condition does not interfere with the person’s capacity to give consent.

Participants, their family member, or legal guardian are advised that they can withdraw from the study at any time and that withdrawal will not jeopardise any treatment or relationship with the hospital. A Revocation of Consent form is provided to all participants on enrolment as a means of withdrawal and clearly states that in the event of participant withdrawal, data already collected will be included in the aggregated analysis but that no further data will be collected.

### Additional consent provisions for collection and use of participant data {26b}

As part of the process evaluation that is undertaken alongside the clinical trial [16], it is important that we are able to determine the proportion of eligible patients who refused to participate and describe the demographic characteristics of *both* eligible patients who participated and those who do not (i.e. non-participants). To achieve this, we are collecting demographic characteristics for non-participants, such as age, gender, type of admission (i.e. medical or surgical), and pressure injury risk. Therefore, as part of the consent procedure (i.e. included as an additional tick box in the PICF), eligible patients or their proxy who do not wish to participate will be invited to consent to some non-identifiable information about them being collected by the research team.

In the latter part of the trial, a subsample of patients from the intervention group and nursing staff will be purposively selected to reflect a range of demographic and clinical factors as part of the process evaluation [16]. Nursing staff will be invited to participate following an informed consent process. Research personnel who are involved in the implementation of the trial (such as ResNs and outcome assessors) will also be invited to participate in individual, semi-structured interviews following an informed consent process. The aim of the interviews will be to explore ResN perceptions and experiences of being involved in the EEPOC trial.

## Interventions

### Explanation for the choice of comparators {6b}

The comparator is routine care as per hospital procedures informed by national standards and international practice guidelines [1, 18].

### Intervention description {11a}

#### Intervention: routine care plus dressing

If allocated to the intervention group, participants have a prophylactic sacral dressing applied by a trained research nurse (ResN), following the manufacturer’s instructions. The trial dressing is the Mepilex® Border Sacrum, a foam border silicon dressing available in two sizes to accommodate physical differences of participants. The dressing manufacturer recommends the dressing be changed when the edge begins to roll and/or loses adhesion or becomes soiled. Dressings will also be changed if saturation occurs, if staff accidentally remove the dressing, or if the dressing becomes dislodged.

The ResN takes a baseline photograph of the participant’s sacrum and then applies a silicone foam border dressing (Mepilex®). The dressing and sacral skin are visually inspected every day for up to 14 days. The frequency of dressing replacements (with reason, including device deficiencies) will be documented. Participants in the intervention group will continue to receive routine care, as per hospital procedures informed by national standards and international practice guidelines [1, 18].

#### Control: routine care

Participants allocated to the control group continue to receive routine care according to the hospital procedures informed by national standards and international practice guidelines [1, 18]. Routine care consists of regular skin inspection and assessment, nursing care via use of a pressure redistribution overlay on a standard mattress, or removal of a standard mattress and replacement with a pressure redistributing mattress, possible multidisciplinary review, and second hourly repositioning.

Participants in the control group also have their sacrum photographed every day following recruitment but do not have a dressing applied to the area. Photographs are edited in the same way as the intervention group before being sent to a blinded-to-intervention assessor for evaluation.

### Criteria for discontinuing or modifying allocated interventions {11b}

#### Intervention

Where there is evidence of skin irritation and/or participant-reported discomfort and/or itchiness due to the dressing, the dressing will be removed, the skin reaction reported using standard hospital incident reporting protocols, and the patient exited from the clinical study. Where the patient has developed persistent urinary and/or faecal incontinence, the sacral dressing will be removed, and the patient exited from the clinical study. In our pilot trial, only six participants (5% of the total sample) reported dressing discomfort, due to dressing migration or mild itchiness without any other clinical symptoms of allergic reaction [13].

Note that in either group, diagnosis of a sacral HAPI resulting in the patient exiting the study will be made on a case-by-case basis in consultation with the clinician caring for the patient and/or the team leader. This decision will not influence the assessment of the outcome assessor (i.e. they will not be aware of the decision made by the clinician/team leader). Where there is indecision among the clinical staff caring for the patient regarding the diagnosis of a Stage 1 PI, the assessment by the blinded assessor will be used to determine if the patient should exit the clinical study.

### Strategies to improve adherence to interventions {11c}

ResNs allocated to each site received training regarding Good Clinical Practice (GCP), the study protocol (including subsequent amendments), intervention, recruitment strategies, data collection using an electronic database, and inspection of the sacrum surgical site including how to take photographs (in terms of consistent angle, lighting, and distance where possible) and edit photographs prior to commencement in the role. A manual detailing the standard operating procedures (SOP) for the trial (including subsequent updates) is available to all members of the research team for top-up training to ensure ongoing protocol adherence and consistency. The information provided ensures all participants receive identical verbal and written information and instructions regarding the study. In addition, ResNs have received education from the dressing manufacturer representatives.

Audits of 10% of photographs have been taken to evaluate the quality of photographs (e.g. lighting, angle, distance) and photo modification to conceal dressing markings. Detailed instructions and images have been added to the SOP to provide continued support to ResNs and training new staff.

Regular assessment of treatment fidelity by the clinical trial coordinator (CTC) focuses on data integrity, the trial dressing and its application, photography, and image editing. Fleiss Kappa analysis will estimate inter-rater reliability between blinded outcome assessors. A score of ≥08 will be considered acceptable.

### Relevant concomitant care permitted or prohibited during the trial {11d}

Routine care according to hospital procedures and informed by state governance and international standards [1, 18] continues throughout the trial for all participants.

### Provisions for post-trial care {30}

Routine care according to hospital procedures informed by state governance and international standards [1, 18] is provided within the public health service in Queensland, Australia.

### Outcomes {12}

#### Outcome measure primary outcome

Cumulative incidence ratio will be calculated as a proportion of the difference between the number of sacral HAPI between the intervention and control groups (i.e. representing relative benefit). The difference in the risk of developing sacral HAPI within 14 days will be used to test the difference between the prophylactic silicone foam dressing and routine care.

#### Secondary outcomes

1. Time to onset of sacral HAPI in days up to 14 days
2. Sacral HAPI incidence rates per 1000 bed days
3. Severity of sacral HAPI up to 14 days, i.e. depth of tissue damage assessed using an internationally and nationally recognised classification system [1, 18]. This classification is based on the depth of skin and tissue damage, from non-blanchable erythema (stage 1) to full thickness tissue loss (stage 4) and unstageable damage and suspected deep tissue injury
4. Cost-effectiveness (an additional 14 days, up to 28 days): via an economic evaluation to compare the healthcare costs of prevention (using the prophylactic sacral dressings in addition to routine care compared to routine care alone) and benefits of prevention in terms of any difference in either incidence or treatment costs of sacral HAPI
5. Process (implementation) evaluation to determine intervention fidelity will allow a better understanding of the hospital contexts in which the trial was implemented. A process evaluation will identify contextual factors and co-interventions related to hospital procedures or individual clinical practice that may operate concurrently with the intervention. Fidelity will assess whether the delivery of the intervention to participants as outlined in the SOP and training processes is consistent (via quantitative monitoring and assessment methods) and explored (via qualitative methods)

### Participant timeline {13}

The participant timeline is shown in Table 1.

**Table 1:** Participant timeline based on the SPIRIT statement [19]

	Study period						
	Enrolment and allocation	Baseline data collection	Daily data collection (post-allocation)		Study exit	Evaluation	Trial close-out
Time point			***Up to 14 days*** *Prevention*	***Up to 28 days***^e^ *Treatment*			
**Enrolment** (within 36 h of hospital admission)							
**Eligibility screen**	X						
**Informed consent**	X						
**Allocation** (randomisation)	X						
**Baseline data collection**							
Hospital information		X					
Participant demographics		X					
Participant health data		X					
PI risk assessment		X					
Photography of the sacrum		X					
Application of dressing (intervention only)		X					
**Intervention**							
Routine care only			X				
Routine care + prophylactic dressing			X				
**Outcome variables**							
Skin assessment^a^			X				
De-identified photograph of the sacrum and photo editing			X				
Dressing change/reason^b^			X				
HAPI			X				
Other complications^c^			X				
**Economic evaluation**							
Hospital length of stay^d^					X		
Dressing used for HAPI			X				
Staff time to apply, remove, and change dressing for prevention (subsample)			X				
Resources used to treat any local adverse effects of dressings for prevention			X				
Other resources used to prevent HAPI			X				
Resources (including dressings) used to treat HAPI				X			
**Process evaluation** [16]							
Context			X	X		X	
Fidelity			X	X		X	
Dose delivered			X	X		X	
Dose received (exposure)			X	X		X	
Recruitment	X	X				X	
Reach	X	X				X	
**Discharge, death, or withdrawal**					X		
***Reports to HREC, funding body, site visits, DSMB meetings***							X^f^

*HAPI* hospital-acquired pressure injury, *HREC* Human Research Ethics Committee, *DSMB* Data Safety Monitoring Board

^a^Participants’ demographic and clinical data, e.g. gender, diagnosis, reason/type of admission, mobility status, body mass index (BMI), nutritional deficiencies, the type of and number of comorbidities, smoking status, PI risk, existing PI (other than sacral) and its treatment/management, previous history of PI, number of days enrolled in the trial, and hospital length of stay, will be recorded in a separate electronic case report form

^b^Dressing change/reason = may include skin itchiness/irritation dressing soiled, rolled-up, saturated, in situ for more than several days or as indicated by clinical practice (as per manufacturer recommendations), or other device deficiency

^c^Other complications = allergic reaction, persistent faecal incontinence, unable to be moved

^d^Hospital length of stay recorded for all trial participants

^e^Only relevant for participants who develop HAPI within the 14-day trial will be followed up until day 28

^f^Reports to HREC, funding body, site visits, and DSMB meetings are also completed at multiple, specific time points during the trial (e.g. annual HREC reports)

### Sample size {14}

The sample size is based on the effect size reported in published research [2]. HAPI cumulative incidence in similar settings ranged from 4 to 13% in The Border Trial [8]. We based calculations on an expected effect of intervention on the primary outcome, calculated using a cumulative incidence ratio of 0.5 (0.46 in our pilot trial) [13], between groups. This assumed a cumulative incidence of 5% in the intervention group compared to 10% in the control group. To obtain 90% power with a two-sided *α* level of 0.05, a total 578 patients per group are required. To allow for attrition, a further 10% will be recruited with a total sample of 1320 (*n* = 220 per group/site).

### Recruitment {15}

Our target sample is 1320 participants (i.e. 220 per group/site) which also accounts for an anticipated 10% lost to follow-up. Originally, the recruitment phase was to be completed in about 3.5 years, dependent upon seasonal fluctuations, hospital bed closures, and holiday periods. However, the COVID-19 pandemic has delayed the trial considerably, hence its extension to December 2023.

A screening tool reflecting inclusion and exclusion criteria to identify potential participants was specifically developed based on our previous research in this area [13]. Based on screening criteria, the ResN approaches the potential participant and invites them to participate via the informed consent process.

## Assignment of interventions: allocation

### Sequence generation {16a}

Following eligibility assessment and consent, participants are randomised to either the dressing group or the routine care only group using a 1:1 ratio, permuted block sizes of four, six, and eight, stratified by hospital and division (i.e. medical or surgical).

### Concealment mechanism {16b}

Randomisation is computer-generated via a secure website and can be accessed via a mobile electronic device.

### Implementation {16c}

ResNs at each site log in to a central randomisation service via a secure website, independent of the trial via a mobile electronic device.

## Assignment of interventions: blinding

### Who will be blinded {17a}

While one investigator (RMW), ResNs, the clinical trial coordinator (CTC), nursing staff, and participants are aware of group allocation, the outcome assessors and research team members (including the trial statistician) are blinded to group allocation and analysis. Given the intervention, it is not feasible for patients, healthcare professionals, and ResN to be blinded to group allocation.

On the day of recruitment, ResNs at each site take high-resolution, de-identified digital photographs (including study number, date, and time) of each participant’s sacrum. All photographs are edited using photographic software to “blur” surrounding skin to ensure atraumatic skin markings from the dressing are not visible, before the photographs are forwarded to a blinded, independent outcome assessor.

The outcome assessors have expertise in identifying PIs and evaluate photographs according to the established international classification system [1]. The outcome assessor allocated on the day makes and discloses their assessment back to the ResNs on the same day as the photograph is taken and provided to the assessor.

### Procedure for unblinding if needed {17b}

There is no requirement for emergency unblinding procedures (refer to the “Who will be blinded {17a}” section).

## Data collection and management

### Plans for assessment and collection of outcomes {18a}

After screening, consent, and randomisation, data are collected directly from the participant or their proxy, and their health record at baseline and then daily for up to 14 days until the trial endpoint (i.e. development of a new sacral HAPI, discharge, or death—whichever occurs first). Participants who develop a HAPI in either arm exit the prevention trial and are followed up for up to 14 days (until discharge or death—whichever comes first) for a total of up to 28 days, to provide data on PI treatment to support the economic evaluation.

Each recruited participant’s hospital information (date of birth, unique record number) is recorded in an eCRF available only to investigator RMW, the CTC, and site ResNs to ensure participant confidentiality. Participants’ demographic and health data including gender, diagnosis or surgery, reason for admission, mobility status, body mass index, nutritional deficiencies, the type of and number of comorbidities (such as cardiovascular disease, diabetes, cognitive impairment, and malnutrition on admission), current smoking status, PI risk, existing PI (other than sacral) and its treatment/management, previous history of PI, number of days enrolled in the trial, and HLOS (documented at discharge for all participants) is recorded in a separate eCRF (electronic clinical research form). A photograph is taken of the patient's sacral skin and sent to the blinded outcome assessor to confirm its diagnosis. A trained CTC monitors recruitment and data collection across all sites and monthly reports are presented to the chief and associate investigators.

As part of the process evaluation, where a patient is unable to provide information for self-report data (e.g. Patient Participation in Pressure Injury Prevention Scale, assessment of satisfaction and comfort), the ResN documents the reason [16]. These data are considered “missing” data for the purpose of analyses, but the reason and proportion of participants who do not have these data due to cognitive impairment, for example, will be reported in reports/publications. For other data that requires participant self-report (e.g. in the instance where weight or height is not documented), the ResN elicits this information from the participant’s proxy if possible or attempts to collect this data in accordance with the protocol and SOP manual (e.g. patient’s medical record, Demi Span, using chair scales on the ward where possible).

Data on resource utilisation associated with prevention are collected including the number of dressings used; personnel time to apply, remove, and change dressings; and any treatment required for any local adverse effects from the dressings. To enable an extended cost-benefit analysis, resources used to treat sacral HAPI are also recorded for those patients that develop a sacral PI during the trial. HLOS is recorded for all trial participants on discharge from hospital.

Digital photography is a novel and practical solution to address blinding. Using study-specific and password-protected iPads, research nurses at each site take high-resolution, de-identified digital photographs of every participant’s sacrum at baseline and each subsequent day they are enrolled in the trial. Photographs are edited using Adobe photo editing software to “blur” surrounding skin to conceal atraumatic skin markings for participants in the intervention group or other markings that may indicate the participant is in the control group (e.g. clothing or bedsheets). Except for the baseline photograph, all modified photos are uploaded into a secure data collection platform and forwarded to a blinded outcome assessor on the same day they are taken. Where a stage 1 (i.e. non-balanceable erythema) is suspected, the patient is be repositioned where possible, the site reassessed after 30 min, and the photograph taken (35). For each photograph, the ResN notifies the blinded outcome assessor as to whether the skin is blanching or non-blanching. The patient exits the clinical study if it is determined they have developed a sacral HAPI which is reported to clinical staff. If the clinical staff caring for the patient diagnose a stage 1 HAPI (even if the blinded assessor did not), the patient is still exited from the study. However, this decision is not disclosed to and therefore does not influence the assessment of the blinded outcome assessor. If the patient has a sacral HAPI, they are included in the estimation of PI treatment costs.

### Plans to promote participant retention and complete follow-up {18b}

Screening patients to assess their risk of PI within 36h of hospital admission and expected hospital length of stay ≥24h following recruitment and informed written consent (in person or via a proxy) increases participant retention and enables at least one outcome assessment (i.e. assessment of a de-identified photograph by a blinded outcome assessor). Given the busy nature of acute clinical settings, ResNs often visit participants several times a day to collect outcome data.

### Data management {19}

Data are entered directly into REDCap (Research Electronic Data Capture), via a secure web platform for building and managing online databases, by ResNs using a password-protected iPad. The eCRF was adapted from the paper version used in our pilot trial [13].

The data management plan for data that will be retained on completion of the trial is as follows:

- All data retained will be in an electronic, re-identifiable format (i.e. stored separately from participant identifying information, with only participants’ unique study number documented in both datasets)
- Paper-based/non-electronic data (e.g. data collection instruments and research materials such as PICFs) will be transferred to an electronic format, and all paper-based/non-electronic data destroyed
- Electronic data (including re-identifiable raw data sets exported from REDCap, electronic photographs, and research materials such as PICFs) will be securely stored using password-protected computers and/or using Griffith University’s secure cloud storage systems. These data will be stored indefinitely for potential future research use by the research team (in accordance with the NHMRC Australian Code for Responsible Conduct of Research)

Data management plans can be accessed by team members of the CTC and ResNs via the trial protocol and SOP.

### Confidentiality {27}

All participants are assigned a study-specific identification number. Exported data are de-identified and stored separately from the data set containing participant identifying information (only the participant’s unique study number is contained in both files for the purpose of re-identification). eCRFs and any study information are stored on a password-protected computer or secure Griffith University applications accessible only by the research team. All paper copies of study information, including the signed participant consent forms, are stored in a locked cupboard, in a secure room, located at the hospital site where the data are collected. All collected paper-based data are securely stored for the duration of the trial and will be transferred to an electronic format, with paper-based/non-electronic data destroyed once the trial has ended. Re-identifiable electronic data will be securely stored indefinitely for scholarly priority (e.g. secondary analysis) and in accordance with the NHMRC Australian Code for the Responsible Conduct of Research and GCP. Individual participant data (IPD) is not shared outside of the research team as data collected may be potentially re-identifiable. This decision was made as IPD sharing may have discouraged potential participants from agreeing to participate if they were aware their data may be shared with others. De-identified electronic data (i.e. name, date of birth and hospital number removed) will be shared with members of the research team who are located at institutions outside of Australia.

### Plans for collection, laboratory evaluation, and storage of biological specimens for genetic or molecular analysis in this trial/future use {33}

N/A. This trial does not collect biological specimens for analysis.

## Statistical methods

### Statistical methods for primary and secondary outcomes {20a}

The patient is the unit of measurement. The primary outcome, development of a new sacral HAPI, is dichotomous. The main analysis will be based on comparisons of the cumulative incidence of pressure injuries in the two groups. Relative risk (RR) of HAPI for the intervention group compared to the control group will be computed and reported along with 95% confidence intervals and *P* values.

We anticipate that prognostic imbalance of known (e.g. age, BMI, number of comorbidities) and unknown prognostic factors is unlikely between the groups due to central randomisation and treatment concealment. However, if we find any differences, we will conduct adjusted analyses using multiple regression models. We do not expect effect sizes to vary between hospitals, but centre-to-centre variability will be assessed, and cluster-adjusted analysis will be carried out as a form of sensitivity analysis if required. Patients’ declining to participate in the trial are given the opportunity to consent to some non-identifiable data being collected (i.e. admission type, age, sex, and PI risk score). These data will enable us to compare some characteristics of eligible and consenting patients (i.e. participants) versus patients who are eligible but not consenting (i.e. non-participants).

For the secondary endpoints, “time to HAPI” will be analysed using the Kaplan-Meier curve and log rank tests. Hazard ratios along with 95% confidence intervals will be reported. Additional Cox regression models will be used to detect the influence of prognostic factors. As for the “stage of HAPI”, a chi-square test will evaluate if the intervention group differs from the control patients. All data will be entered directly into a web-based data collection tool (REDCap) and then exported into SPSS (V 24). Prior to analysis, a rigorous process of data cleaning to check outlying figures, missing, and implausible data against source data will be undertaken. Sample attrition will be managed via intention-to-treat analysis to ensure an unbiased comparison of the groups by randomisation. An expert biostatistician will lead all analyses.

A parallel economic evaluation from the health system perspective using patient-level data from the trial to compare costs and effects of prophylactic silicone foam sacral dressings in addition to routine care with routine care alone will be undertaken. In the first step, a cost-effectiveness analysis will compare the costs and effects of prevention using the trial primary outcome (cumulative incidence) as the measure of effect. Data collected on costs and clinical outcomes will be used to calculate the incremental cost per sacral HAPI avoided, which is the difference in the mean costs of prevention (i.e. measured to the trial endpoint) divided by the difference in sacral HAPI incidence. In an extended step, a cost-benefit analysis will be undertaken to incorporate the benefits of prevention measured in dollars using data from the extended 28-day follow-up. Benefits will include any difference in costs of sacral HAPI treatment and HLOS between groups (documented on discharge for all participants).

As already described, a parallel process evaluation is being undertaken alongside the trial [16, 17].

### Interim analyses {21b}

An interim analysis will be undertaken by multidisciplinary Data Safety Monitoring Board (DSMB) midway through the trial.

Additional ad hoc meetings may be scheduled if requested by either the CIs or the DSMB.

### Methods for additional analyses (e.g. subgroup analyses) {20b}

Post hoc subgroup analyses may be undertaken where there are differences between groups in relation to demographic and/or clinical characteristics.

### Methods in analysis to handle protocol non-adherence and any statistical methods to handle missing data {20c}

All randomised patient data will be analysed by intention-to-treat, regardless of treatment received. However, a per-protocol analysis for trial participants who consented, were randomised, completed the baseline assessment, and had at least one outcome assessment will also be conducted. Where enrolled participants do not have at least one outcome assessment (due to for example unexpected early discharge), they will be defined as “lost to follow-up”, regardless of their time in the trial. While all efforts are made to reduce loss to follow-up, as in most trials, it is inevitable that some missing outcome data may occur. In such circumstances, we envisage using best (no PI) and worst case (PI) scenarios to analyse the impact of missing outcome. These scenarios will form sensitivity analyses to assess the impact of missing outcome data, assuming those missing outcomes had no event or had an event.

### Plans to give access to the full protocol, participant-level data, and statistical code {31c}

This document is the full protocol. Anyone interested in other data or documentation should contact the corresponding author.

## Oversight and monitoring

### Composition of the coordinating centre and trial steering committee {5d}

Day-to-day trial activities are coordinated by the CTC who is in continuous communication with the ResNs and investigator RMW. The wider team comprising all chief investigators, associate investigators from each participating site, and the CTC meet monthly and will continue to do so over the course of the trial.

### Composition of the data monitoring committee, its role, and reporting structure {21a}

Informed by the National Health and Medical Research Council guidance for Safety monitoring and reporting in clinical trials involving therapeutic goods [20], and independent of trial investigators, a DSMB with expertise in clinical trials, epidemiology, biostatistics, and nursing will review accumulating safety data after 50% of participants are randomised. Members of the DSMB will be unblinded to group allocation. Their review will be reported to the Human Research Ethics Committees. Where relevant, serious adverse event data will also be reported to the Australian Therapeutic Goods Administration (TGA) [20].

### Adverse event reporting and harms {22}

Serious adverse events associated with the intervention will be reported to the HREC. The CTC, chief investigators, and site associate investigators (AI) are responsible for:

- Collating safety and adverse event information and submitting annual safety reports to Human Research Ethics Committees (HREC)
- Reporting expected and unexpected adverse events (AE) to the DSMB
- Reporting to the institution without undue delay and no later than 72 h after becoming aware of all significant safety issues and unanticipated serious adverse device effects (USADEs) occurring at the site
- Maintaining detailed, up-to-date records of all adverse device effects (ADEs) and device deficiencies that ResNs report
- Communicating safety information to investigators and/or HRECs, to clarify the impact of each report on patient safety, trial conduct, and/or trial documentation
- Assessing and categorising information received from investigators and reporting all USADEs occurring in participants to the TGA in accordance with NHMRC guidelines

### Frequency and plans for auditing trial conduct {23}

As outlined throughout this protocol, the CTC monitors all aspects of the trial on a weekly basis. This includes adherence to the protocol, ethics and governance, management of databases, outcomes assessments, photo auditing, training of research staff, and regular reporting to CIs.

Individual sites are also monitored by their local health service governance structures.

### Plans for communicating important protocol amendments to relevant parties (e.g. trial participants, ethical committees) {25}

All key amendments to the protocol and PICFs are submitted to the overarching HREC for approval and site health service governance authorities notified.

### Dissemination plans {31a}

Results will be presented in aggregate form to ensure personal information is non-identifiable. We will present results at the local hospitals and other fora and prepare a succinct non-technical paper discussing relevance of findings, application to practice and recommendations for future research for dissemination to various media and professional groups. Conference abstracts will be submitted to major international meetings of wound prevention and treatment, nursing, and medical groups. Trial results will be published in high-impact generalist journals. Aggregated results will also be given to participants upon request.

## Discussion

This protocol outlines the design of this pragmatic RCT to examine the effect of prophylactic sacral dressings on the cumulative incidence of PI in hospitalised adults. Our trial has several strengths. We are using an appropriately powered, robust multicentre pragmatic RCT design that is the largest trial of its kinds in this area. We are conducting an economic evaluation alongside the trial, and a process evaluation to ensure any difference in outcomes between the groups can be attributable to the intervention or contextual issues. With so few economic studies in this area, this parallel cost-effectiveness analysis will provide valuable health economic information to guide decision-making of health service managers to better balance quality patient care with economic efficiencies [21]. Digital photography and photo editing software are being used, which enables blinded, independent outcome assessors to assess the sacral skin for PI remotely, thereby reducing their ability to identify group allocation. This method represents a significant and innovative approach to clinical research. Finally, this multicentre RCT is independent of industry funding and therefore minimises potential for a conflict of interest and bias.

## Trial status

Recruitment commenced on July 10, 2020, and continues with an approved end date of December 31, 2023, due to the COVID-19 pandemic.

This current protocol is version 6, dated 15/10/2021.

## Data Availability

Data may be available for collaborators on request to the corresponding author, Associate Professor Rachel M Walker r.walker@griffith.edu.au.

## Abbreviations

ADE: Adverse device effect
AE: Adverse event
APuP: Attitudes toward Pressure ulcer Prevention [instrument]
CTC: Clinical trial coordinator
DSMB: Data Safety Monitoring Board
eCRF: Electronic clinical research form
GCP: Good Clinical Practice
HAPI: Hospital-acquired pressure injury
HREC: Human Research Ethics Committee
IPD: Individual participant data
NHMRC: National Health and Medical Research Council [Australia]
PI: Pressure injury
PICF: Participant information and consent form
REDCap: Research Electronic Data Capture
ResN: Research nurse
SEM: Sub-epidermal moisture [scanner]
SOP: Standard operating procedures
TGA: Therapeutic Goods Administration [Australia]
USADE: Unanticipated serious adverse device effect
